# Gut commensal *Parabacteroides goldsteinii*-derived CDCA mediates FXR signalling to improve mucosal barrier function

**DOI:** 10.1038/s41522-026-01035-2

**Published:** 2026-06-06

**Authors:** Jiantao Yang, Kailong Qin, Qianggang Wang, Wei Guo, Xiaojun Yang

**Affiliations:** 1 College of Animal Science and Technology, Northwest A&F University, Yangling, Shaanxi China; 2 Jiangsu Institute of Poultry Science, Yangzhou, Jiangsu China

**Keywords:** Gastroenterology, Microbiology

## Abstract

The gut microbiome plays a crucial role in maintaining barrier integrity. However, it remains urgently needed to mining the gut microbiota that contribute to intestinal mucus barrier and to understand their biological mechanisms. In this study, the Lueyang black-boned chicken (LBC, a Chinese native breed) exhibited higher abundances of Bacteroidetes and *Parabacteroides* and stronger intestinal barrier function than Arbor Acres (AA, a commercial breed) broilers. Microbiota transplantation from LBC to AA broilers improved the intestinal barrier function. *Bacteroides* expanded the exclusive metabolic niche of *Parabacteroides goldsteinii* by producing small metabolites, thereby increasing *P. goldsteinii* abundance within the gut. *P. goldsteinii* was identified as the main contributor to the enhanced intestinal mucosal barrier. *P. goldsteinii* bio-transformed the taurochenodeoxycholic acid into chenodeoxycholic acid, promoting mucin production via farnesoid X receptor activation. Collectively, these findings highlight that the gut microbiota of LBC creates an exclusive metabolic niche for *P. goldsteinii* through cross-feeding, and reveal the critical role of *P. goldsteinii*-mediated bile acid biotransformation in enhancing intestinal mucosal barrier function.

## Introduction

The intestinal mucosal barrier, located at the interface between the gut microbiota and intestinal epithelium, provides the first line of defence against pathogens and antigens and plays a vital role in homeostasis^1,2^. Barrier dysfunction is implicated in various human diseases, including inflammatory bowel disease (IBD), celiac disease, and metabolic syndrome^3,4^. Intestinal mucins, secreted by goblet cells, are a key protein component of this barrier^5,6^. Impaired mucin production reduces mucus layer thickness and increase permeability, facilitating translocation of pathogens and endotoxins^7^. Therefore, there is an urgent need for new strategies to improve the early development of the intestinal mucosal barrier.

The gut microbiota, a complex and dynamic microbial ecosystem, plays a crucial role in maintaining the intestinal mucus barrier function^1,8^. Notably, germ-free mice have fewer goblet cells and exhibit different barrier properties compared to conventionally raised mice^9^. Several commensals-including *Lachnospiraceae*, *Ruminococcaceae*, and *Akkermansia muciniphila*-have been associated with enhanced barrier function^10^. Within the microbial ecosystem, cross-feeding interactions among bacteria regulate community structure and function^11,12^. For example, *Bacteroides vulgatus* produces *N*-acetylglucosamine that promotes *Akkermansia muciniphila* colonization through cross-feeding, protecting against diet-induced intestinal damage^13^. Moreover, gut microbiota participate in bile acids biotransformation and secondary bile acids regulate the barrier function through the activation of farnesoid X receptor (FXR) and Takeda G-protein receptor (TGR5) signaling^14^. For instance, chenodeoxycholic acid (CDCA) protects against lipopolysaccharide-induced intestinal barrier dysfunction via FXR activation^15^. However, the specific microbial strains that modulate bile acid composition to enhance barrier function remain largely unknown.

Native breeds of poultry possess a rich reservoir of microbial resources associated with disease resistance and stress tolerance^16–18^. In this study, the intestinal barrier function and gut microbial landscape of Lueyang black-boned chickens (LBC, a Chinese native breed) and Arbor Acres (AA) commercial chickens was profiled using tissue morphology, metagenomic sequencing and 16S rRNA gene sequencing. We found that microbiota transplantation (MT) from LBC to AA chickens enhanced intestinal mucosal barrier function. We identified *P. goldsteinii* as a key commensal that enhances mucosal barrier function through TCDCA-to-CDCA biotransformation and subsequent FXR activation. Additionally, in vitro experimental results demonstrate that the gut microbiota of LBC creates an exclusive metabolic niche enables *P. goldsteinii* growth through cross-feeding.

## Results

### Intestinal mucosal barrier differs significantly between LBC and AA broilers

**Fig. 1:**
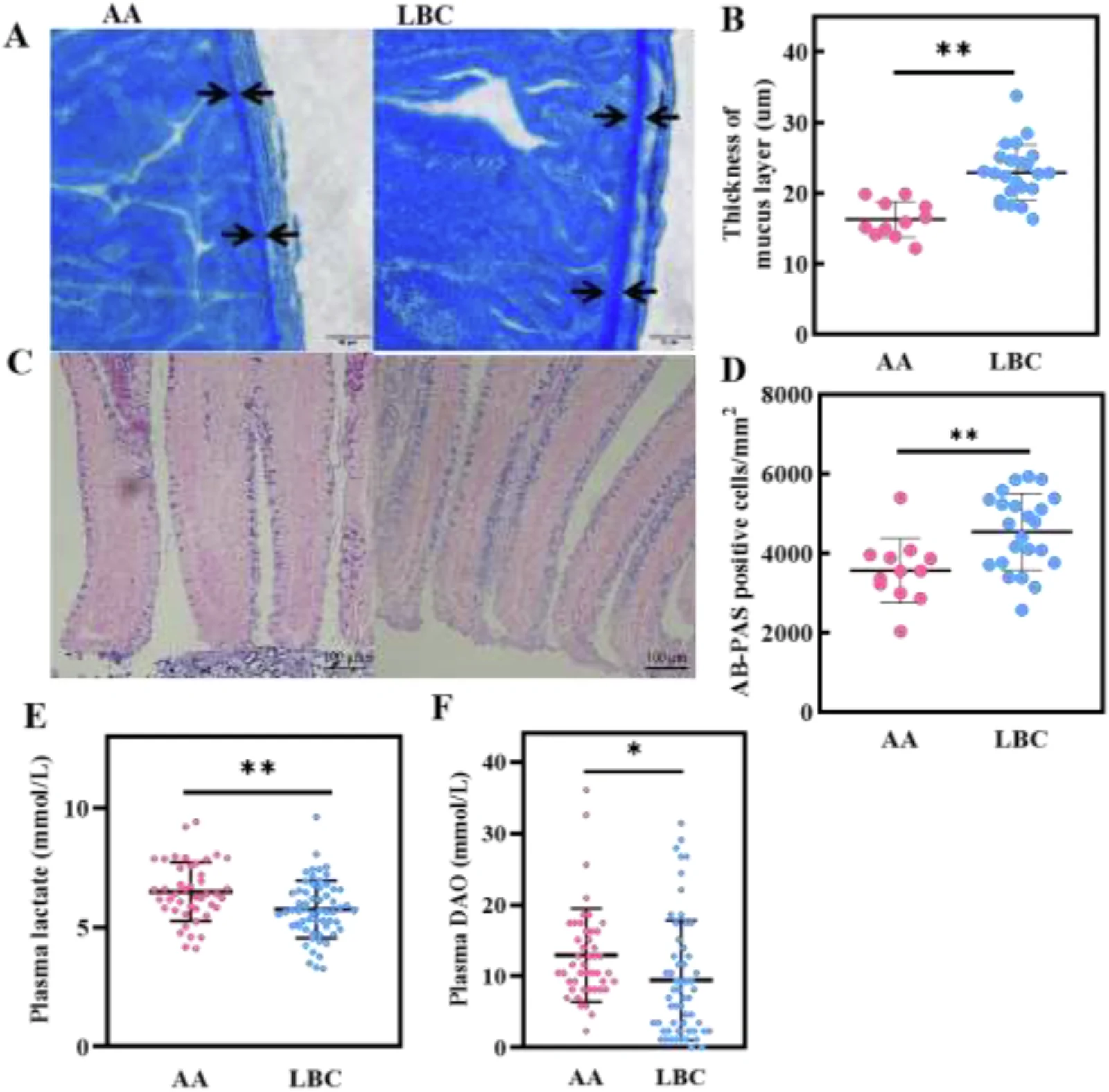
Analysis of the difference of intestinal mucus barrier between LBC and AA broilers. **A**, **B** Representative images of Alcian blue-stained ileal sections, and the thickness of mucus layer. **C**, **D** Representative images of AB-PAS-stained ileal sections, and the number of mucin. **E** Plasma D-lactate levels, (**F**) Plasma DOA. The data are shown as the means ± SEM. Statistical analysis was performed using two-tailed unpaired Student’s *t* tests. *n*  = 12 (AA group in **A**–**D**) and *n*  = 23 (LBC group in **A**–**D**); *n*  = 44 (AA group in **E**, **F**) and *n*  = 60 (LBC group in **E**, **F**).

To compare the intestinal mucosal barrier functions of LBC and AA broilers, tissue morphology and intestinal permeability were analysed. The thickness of the mucus layer and number of mucins per unit area of villi were higher in LBC than in AA broilers (Fig. 1A-D). Plasma d-lactate levels and diamine oxidase activity (DOA) (two key indicators of intestinal epithelial barrier function) were lower in LBC than in AA broilers (Fig. 1E, F).

### A landscape of LBC gut microbiome revealed by metagenomic and 16S rRNA gene amplicon sequencing

**Fig. 2:**
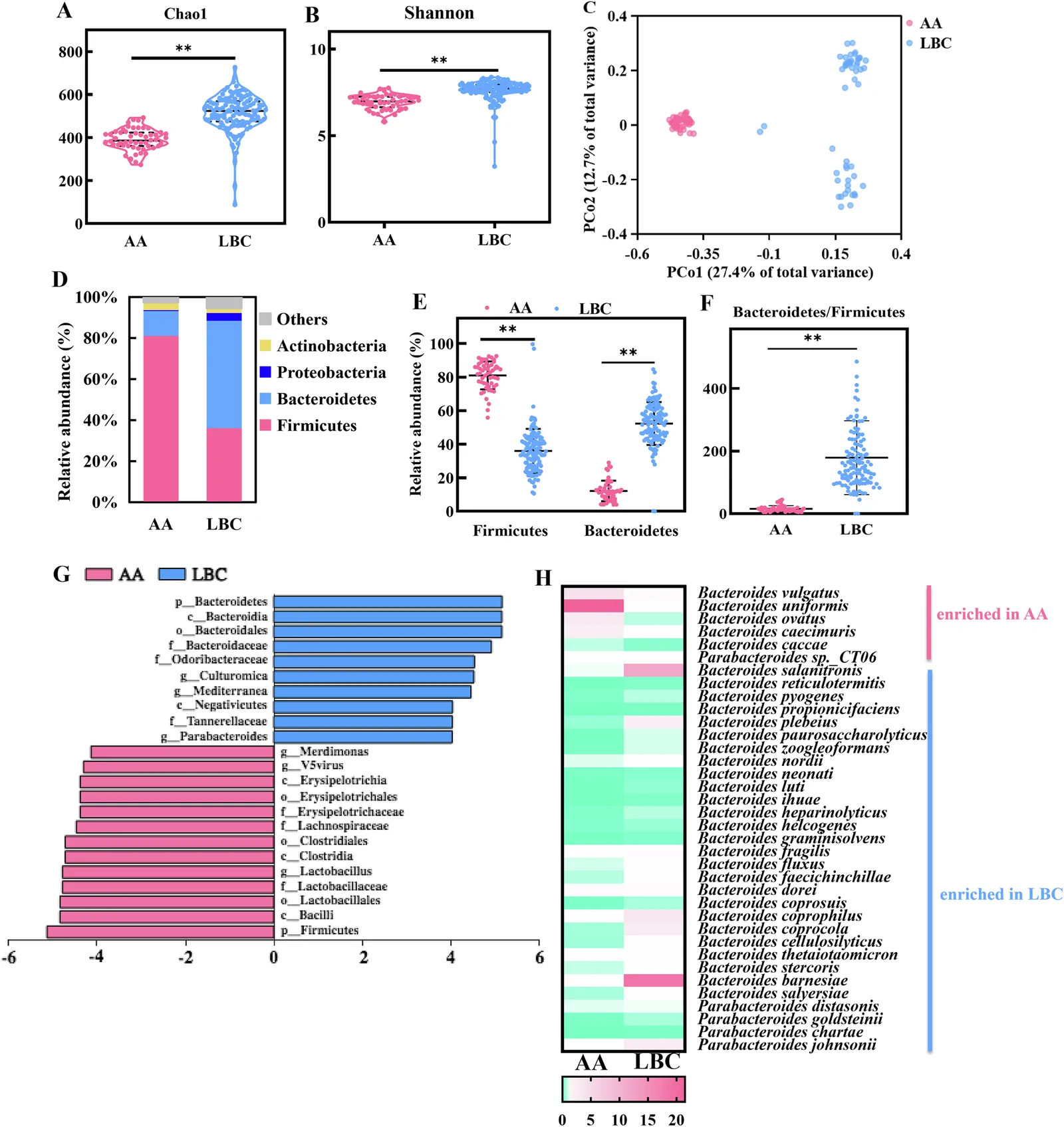
The landscape of LBC gut microbiome. **A**, **B** Analysis of α-diversity index of richness (Chao) and community diversity (Shannon) revealed by 16S rRNA (*n*_AA_ = 54, *n*_LBC_ = 122). **C** PCoA of the gut microbiome composition on the OTU level in chickens revealed by 16S rRNA. **D** Relative abundance of gut bacterial phylum among groups revealed by 16S rRNA. **E** Relative abundance of Firmicutes and Bacteroidetes revealed by 16S rRNA. **F** The ratio of Firmicutes to Bacteroidetes revealed by 16S rRNA. **G** The most differentially abundant taxa at the genus level were identified through LEfSe analysis based on metagenomic sequencing data. **H** The relative abundance of gut bacterial species belonging to the genera *Bacteroides* and *Parabacteroides* among groups revealed by metagenomic sequencing. The data are shown as the mean ± SEM and were evaluated by Kruskal-Wallis test. *n*  = 54 (AA group in **A**–**F**) and *n*  = 122 (LBC group in **A**–**F**); *n*  = 10 (AA group in **G**, **H**) and *n*  = 24 (LBC group in **G**, **H**).

To decipher the gut bacterial community, we characterized the caecal bacterial communities of LBC and AA chickens using 16S rRNA amplicon sequencing. Alpha diversity, based on the Chao1 and Shannon indices, showed that native LBC had significantly higher alpha diversity than commercial AA chickens (Fig. 2A, B). Principal coordinate analysis (PCoA) of beta diversity further highlighted that obvious difference in microbiota communities between LBC and AA chicken (Fig. 2C). At the phylum level, Firmicutes and Bacteroidetes was dominant phylum in the cecal contents in LBC and AA chicken accounted for over 90% relative abundance (Fig. 2D). Moreover, the relative abundances of Bacteroidetes and Bacteroidetes/Firmicutes were higher in LBC than in AA chickens (Fig. 2E, F).

We further used the metagenomics sequencing to mine the potential probiotics that contribute to the intestinal mucus barrier of LBC. LEfSe analysis indicated differences in bacterial taxonomic compositions between LBC and AA (Fig. 2G). LEfSe analysis indicated that gut bacterial communities in the native LBC were mainly enriched with these genera, such as *Culturomica*, *Mediterranea*, and *Parabacteroides* (Fig. 2G). Based on the predominant colonization of the Bacteroidetes in the caecum of the LBC at the phylum level, we analysed the species of the differential genera *Bacteroides* and *Parabacteroides* (phylum Bacteroidetes). Notably, many bacterial species, such as *Bacteroides zoogleoformans*, *B. vulgatus*, *Bacteroides thetaiotaomicron*, *Parabacteroides johnsonii*, and *P. goldsteinii*, were more abundant in the caecum of LBC than AA broilers (Fig. 2H).

Microbial community structure is crucial in determining the functionality of the microbiome. Gram-positive Firmicutes and gram-negative Bacteroidetes possess different strategies for foraging complex carbohydrates^19,20^. KEGG analysis indicated that the gut microbiome in the LBC was mainly enriched in pathways related to glycan biosynthesis and metabolism (Supplementary Fig S1A). However, the enriched pathways in AA chickens were related to ABC transporters and the phosphotransferase system (PTS) (Supplementary Fig S1A). Further analysis of polysaccharide biosynthesis and metabolism showed that the *N*-glycan biosynthesis and other glycan degradation were significantly enriched in LBC compared to that in AA chickens (Supplementary Fig S1B). However, various types of *N*-glycan biosynthesis and glycosaminoglycan degradation were significantly enriched in AA chickens (Supplementary Fig S1B). An increase in the utilization of animal-derived polysaccharides is often associated with the increased utilization of intestinal mucins, dysbiosis of the mucosal barrier, and increased permeability^21,22^. Therefore, further analyses were conducted on pathways related to the utilization of animal-derived polysaccharides. Compared to those in AA broilers, the pathways for dermatan sulfate, chondroitin sulfate, and heparan sulfate degradation were downregulated in the LBC, whereas the pathway for keratan sulfate degradation was upregulated (Supplementary Fig S1C). The KEGG Orthology (KO) level results were consistent with the PTS results, showing that the abundance of caecal microbiota transporters for sucrose and disaccharides (such as K02810, K02796, K02795, K02794, K02770, K02761, K02760, K02759) was significantly higher in AA chickens than in LBC (Supplementary Fig S1D and Table S3).

### Microbiota transplantation from LBC to AA chicken promotes intestinal mucosal barrier function

We conducted an antibiotic-induced gut microbiota disruption model to investigate whether the gut microbiota is associated with the intestinal mucus barrier. The results indicated that the antibiotic treatment disrupted the community structure and reduced the richness and diversity of the cecal microbiota (Supplementary Fig S2A,B). At the phylum level, Bacteroidetes were reduced, while Proteobacteria were increased after antibiotic treatment (Supplementary Fig S2C). At the genus level, the abundances of *Oscillospira*, *Faecalibacterium*, *Lactobacillus*, *Ruminococcaceae_Ruminococcus*, *Ruminococcus*, *Butyricicoccus*, *Blautia*, and *Bacteroides* were reduced (Supplementary Fig S2D). Furthermore, the thickness of the ileal mucus layer and amount of mucin were significantly reduced (Supplementary Fig S3A–D). The levels of fluorescein isothiocyanate-dextran (FITC-D) and D-lactate in the serum were significantly increased (Supplementary Fig S3E, F). These results collectively suggest the potential possibility for improving intestinal mucus barrier function through the modulation of gut microbiota.

**Fig. 3:**
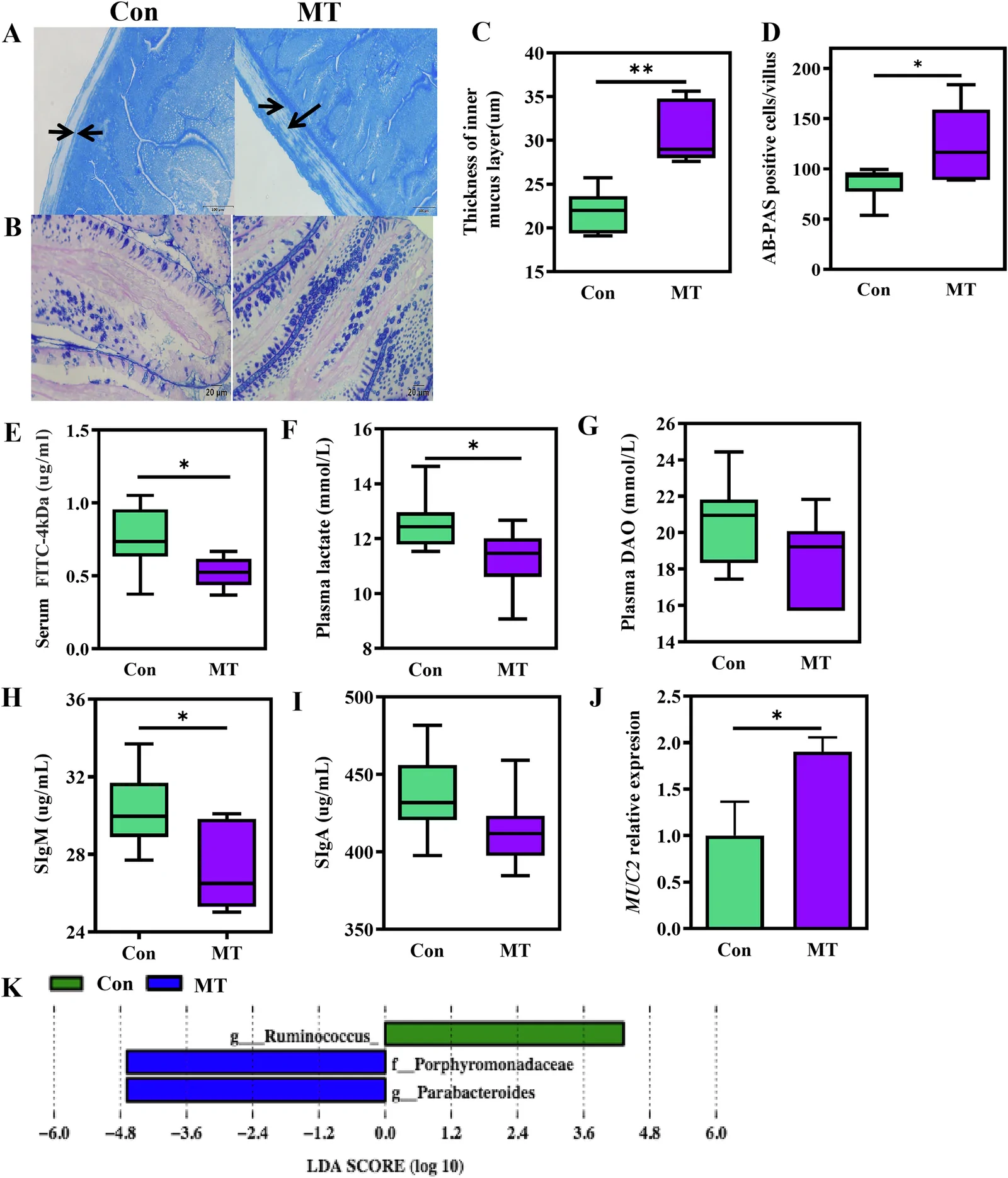
Effects of microbiota transplantation from LBC to AA broilers on intestinal mucosal barrier function. **A**, **C** Representative images of Alcian blue-stained ileal sections, and the thickness of mucus layer. **B**, **D** Representative images of AB-PAS-stained ileal sections, and the number of mucin. **E**–**G** Intestinal permeability serum FITC-dextran level, plasma d-lactic acid level, and plasma DOA, respectively. **H**, **I** Levels of serum SIgM and SIgA. **J** The mRNA levels of *MUC2*. **K** The most differentially abundant taxa at the genus level were identified through LEfSe analysis based on 16S rRNA. Statistical analysis was performed using two-tailed unpaired Student’s *t* tests (*n* = 7).

Next, we investigated whether the gut microbiota of LBC play a crucial role in improving the intestinal mucus barrier function in AA chickens by performing a microbiota transplant (MT). The MT increased the secretion of mucin and thickness of the mucus layer (Fig. 3A–D), but decreased the levels of FITC-D and D-lactate in the serum (Fig. 3E, F). Moreover, the SIgM content was reduced, and *MUC2* expression increased in the ileal mucosa (Fig. 3H, J). The plasma levels of DOA, and the content of SIgA showed no significant differences (Fig. 3G, I).

To further investigate the key commensal bacteria in the LBC that enhance the intestinal mucosal barrier function, 16S rRNA sequencing was performed on the cecal microbiota. Although there was no significant difference in richness and diversity after MT treatment, the community structure showed obvious distinction (Supplementary Fig S4A, B). At the genus level, *Bacteroides*, *Unspecified_Bacteroidales*, *Unspecified_Clostridiales*, *Unspecified_Lachnospiraceae*, *Oscillospira*, and *Parabacteroides* predominated (Supplementary Fig S4C). LEfSe analysis indicated that *Parabacteroides* were the dominant genus in the MT group (Fig. 3K). This is consistent with its higher relative abundance in the LBC, suggesting that *Parabacteroides* may be a key mediator of intestinal mucosal barrier function.

### Gut commensal *P. goldsteinii* contributes to intestinal mucosal barrier function

**Fig. 4:**
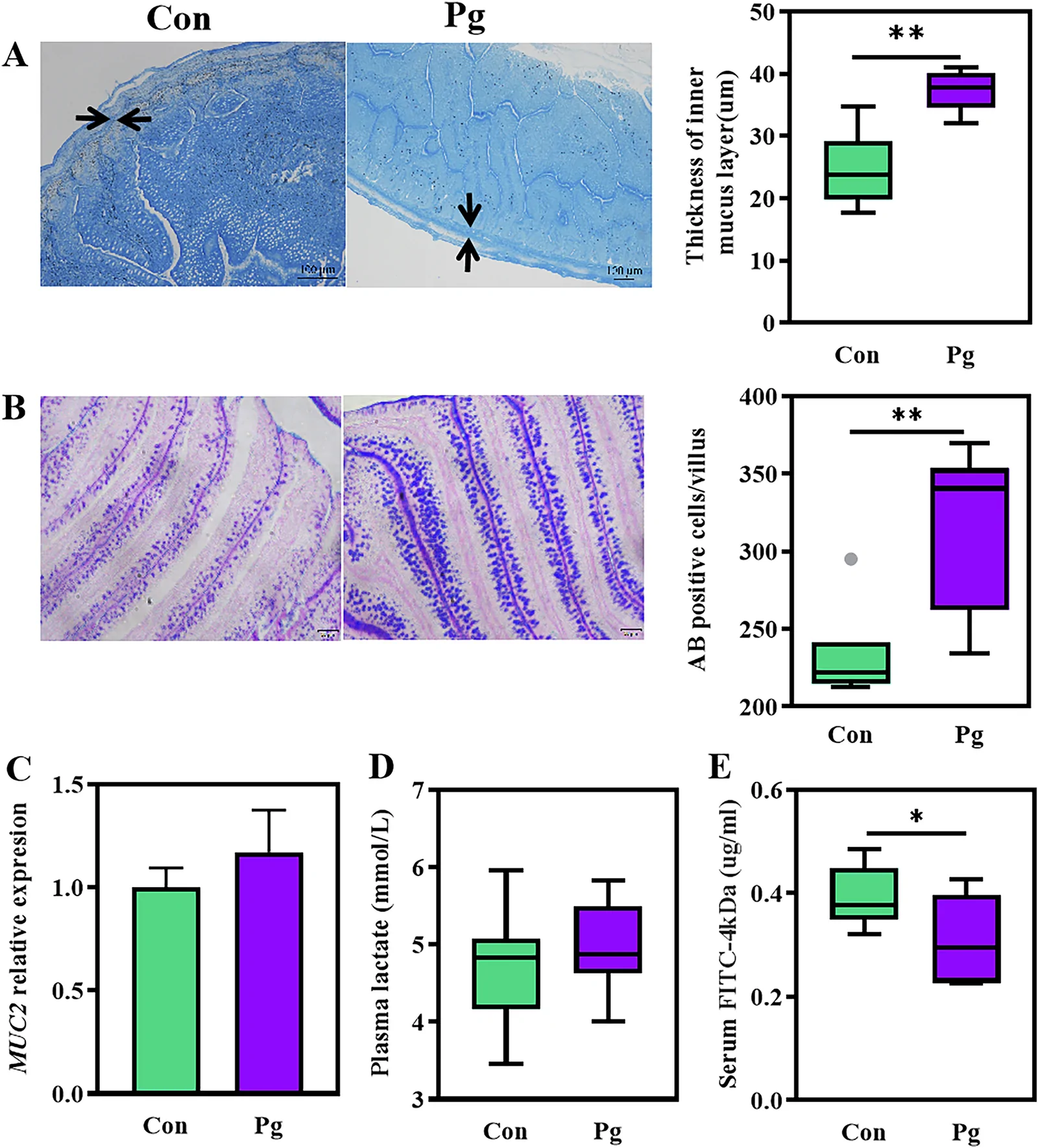
The oral administration of *P. goldsteinii* facilitates the intestinal mucosal barrier function. **A** Representative images of Alcian blue-stained ileal sections, and the thickness of mucus layer. **B** Representative images of AB-PAS-stained ileal sections, and the number of mucin. **C** The mRNA levels of *MUC2*. **D** Plasma d-lactic acid levels. **E** Serum FITC-dextran levels. The data are shown as the means ± SEM. Statistical analysis was performed using two-tailed unpaired Student’s *t* tests (*n* = 10).

Based on the metagenomic of the caecal microbiota in the LBC and 16S rRNA gene sequencing ASVs data of AA broilers from the MT experiment, we analysed the candidate *Parabacteroides* associated with the intestinal mucosal barrier. We identified *P. goldsteinii* may be the greatest contributor. We assessed its role in 1-day-old chicks orally administered *P. goldsteinii* daily for 3 weeks. *P. goldsteinii* promoted intestinal mucosal barrier function by increasing mucin secretion and mucus layer thickness (Fig. 4A, B). Although the *MUC2* expression, and plasma content of lactate were not affected (Fig. 4C, D), the serum level of FITC-D was significantly reduced (Fig. 4E).

### Gut commensal *P. goldsteinii* enhances intestinal mucosal barrier function involved in bile acid biotransformation

**Fig. 5:**
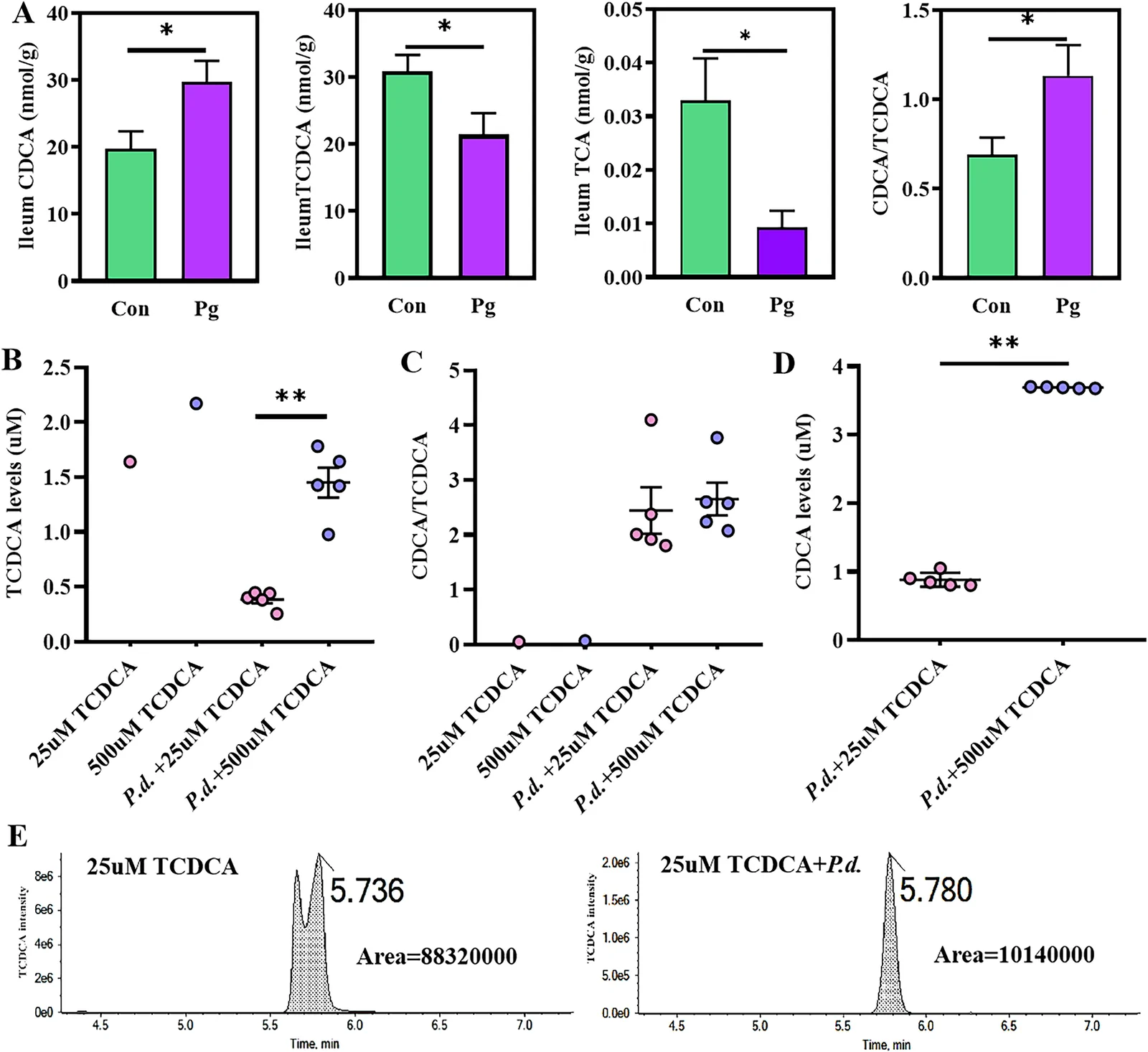
*P. goldsteinii* bio-transforms TCDCA into CDCA. **A** The concentrations of bile acid in the ileal contents. **B** The concentrations of TCDCA in the medium. **C** The ratio of CDCA to TCDCA. **D** The concentrations of CDCA in the medium. **E** The mass spectrometry profile illustrating the changes in TCDCA concentration. The data are shown as the means ± SEM. For (**A**) (*n* = 10), statistical analysis was performed using two-tailed unpaired Student’s *t* tests. For (**B**–**E**) (*n* = 5), the data were analyzed using the Kruskal-Wallis test with Dunn’s multiple comparisons test for comparing groups.

We performed bile acid profiling to identify the metabolites involved in *P. goldsteinii*-mediated intestinal mucus function. Oral administration of *P. goldsteinii* increased the content of CDCA but decreased the levels of TCDCA and TCA in the ileum (Fig. 5A) (Supplementary Fig S5). Additionally, oral administration of *P. goldsteinii* increased the CDCA/TCDCA ratio (Fig. 5A). In addition, the MT from LBC to AA significantly increased the CDCA content in the ileum (Supplementary Fig S6). Antibiotic-induced microbial dysbiosis significantly reduced the CDCA content in the ileum while increasing the TCDCA content (Supplementary Fig S7).

We confirmed whether *P. goldsteinii* bio-transforms TCDCA into CDCA using an in vitro culture in brain–heart infusion (BHI) medium supplemented with TCDCA. As expected, *P. goldsteinii* significantly decreased the TCDCA levels and increased the CDCA/TCDCA ratios by transforming TCDCA into CDCA under anaerobic conditions (Fig. 5B, C). The CDCA levels increased further with higher TCDCA levels (Fig. 5D, E). These results collectively suggested that the biotransformation of CDCA by *P. goldsteinii* may be involved in its enhancement of intestinal mucosal barrier function.

**Fig. 6:**
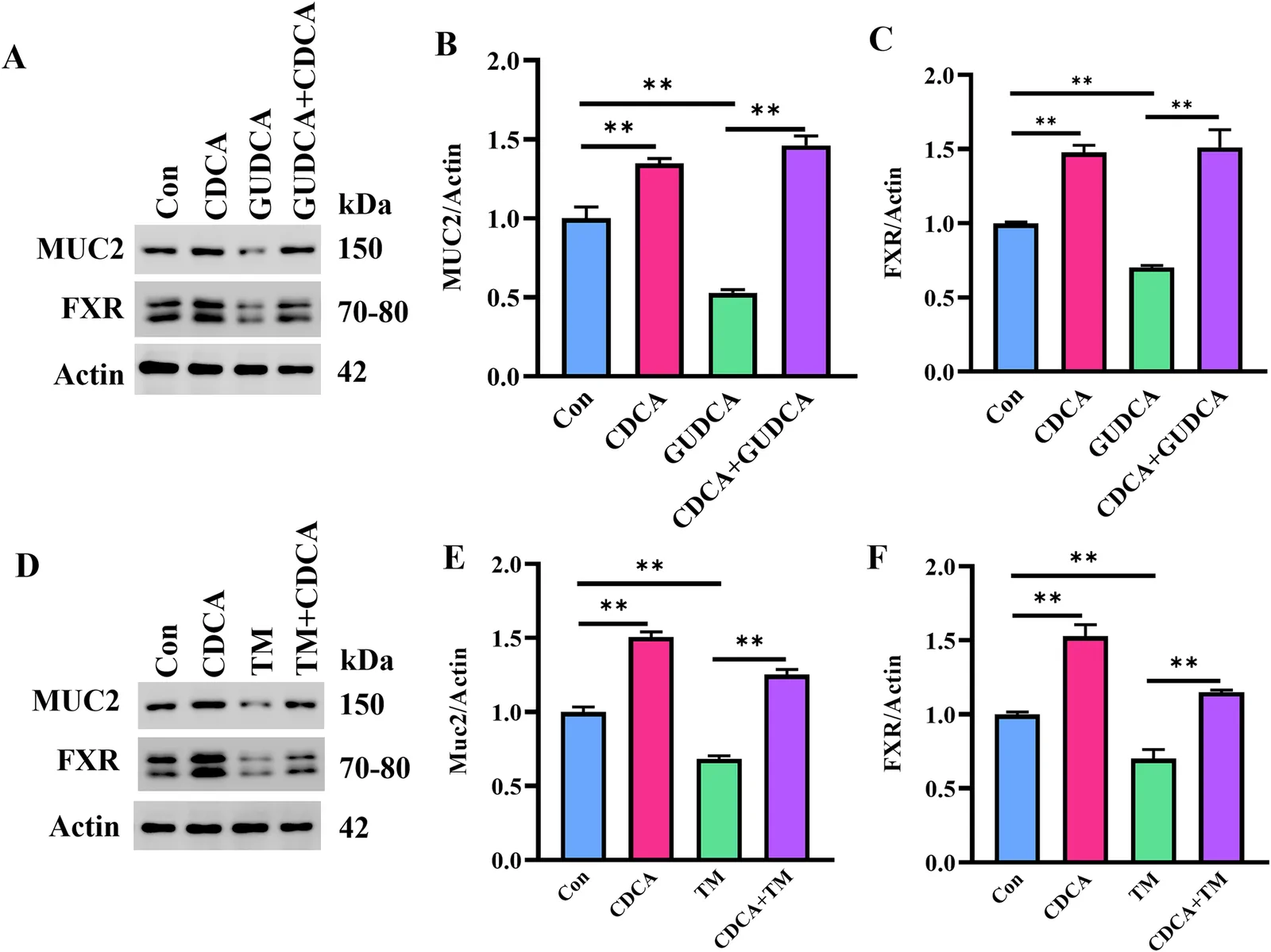
CDCA confers the beneficial effects on the intestinal mucosal barrier function. **A** Representative western blotting results of MUC2 and FXR under CDCA treatment and CDCA treatment with a CDCA inhibitor. **B** Quantitation of the MUC2 levels under CDCA treatment and CDCA treatment with a CDCA inhibitor. **C** Quantitation of the FXR levels under CDCA treatment and CDCA treatment with a CDCA inhibitor. **D** Representative western blotting of MUC2 and FXR under CDCA treatment and CDCA treatment with an FXR inhibitor. **E** Quantitation of the MUC2 levels under under CDCA treatment and CDCA treatment with an FXR inhibitor. **F** Quantitation of the FXR levels under under CDCA treatment and CDCA treatment with an FXR inhibitor. The data are shown as the means ± SEM. the data were analyzed using the Kruskal-Wallis test with Dunn’s multiple comparisons test for comparing groups. (*n* = 3).

Based on the role of microbiota-derived metabolites in modulating the intestinal mucosal barrier, we hypothesized that CDCA may have a direct impact on intestinal epithelial cell function. Given that FXR signaling may be involved in the regulatory roles of bile acids, we examined the activation of FXR signaling by CDCA and its role in intestinal mucosal barrier function. Western blotting confirmed that CDCA activated FXR and increased MUC2 expression (Fig. 6A, B). Inhibition of FXR expression abrogated the effect of CDCA on MUC2 expression (Fig. 6A, C). To further verify the role of CDCA, we inhibited mucin secretion using tunicamycin, a protein glycosylation inhibitor, and found that MUC2 and FXR expression were significantly reduced, whereas CDCA treatment reactivated FXR and increased MUC2 expression (Fig. 6D-F). Together, these findings revealed that the biotransformation of TCDCA into CDCA by *P. goldsteinii* promotes intestinal mucosal barrier function through FXR signaling.

### Enrichment of gut commensal *P. goldsteinii* is associated with cross-feeding by *Bacteroides*

**Fig. 7:**
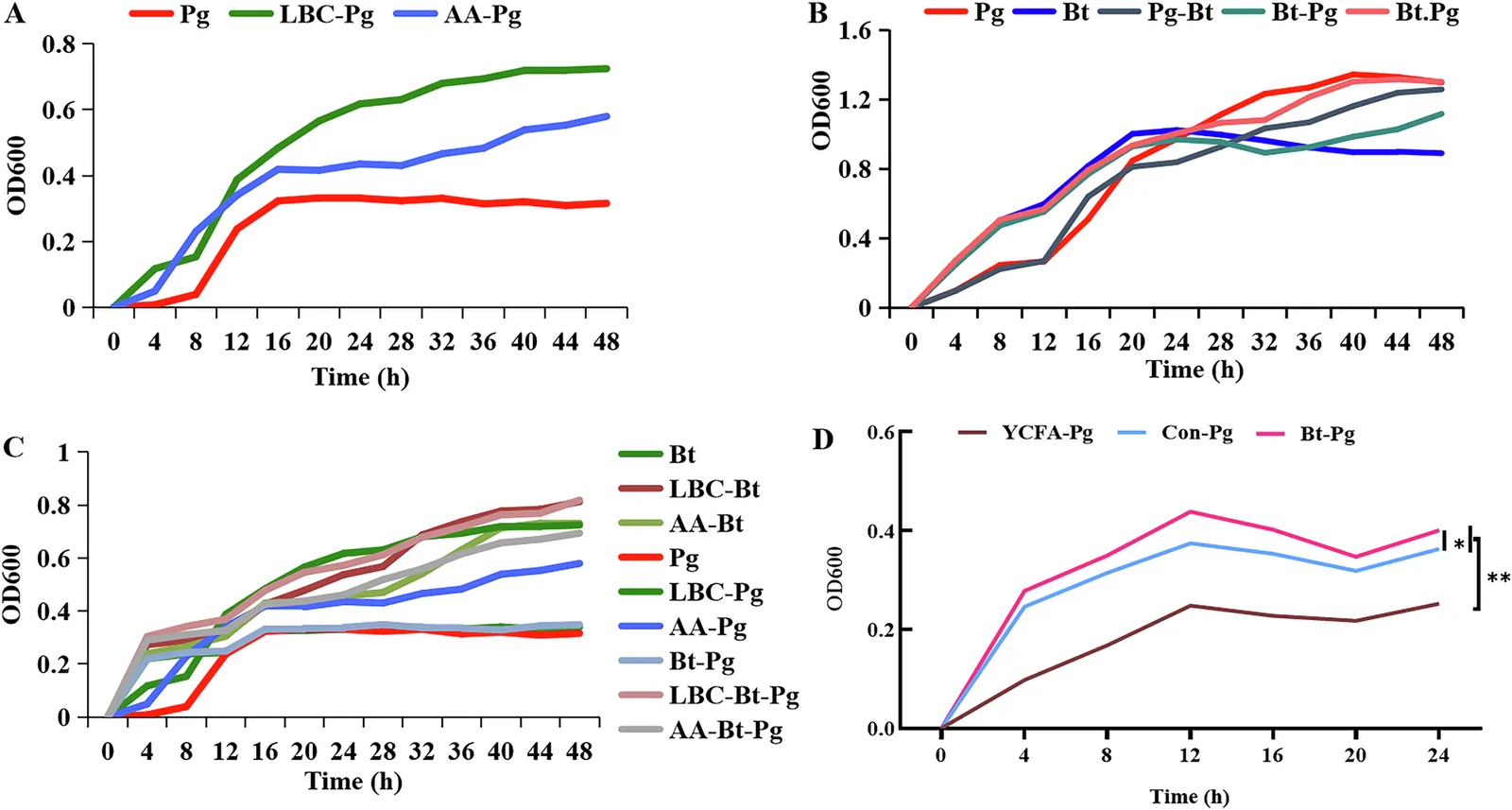
LBC provides an exclusive colonization niche for *P. goldsteinii.* **A** The growth curve of *P. goldsteinii* in YCFA medium supplemented with cecal small-molecule metabolites from LBC and AA. **B** The growth curve of *P. goldsteinii* and *B. thetaiotaomicron* in BHI medium. **C** The growth curve of *P. goldsteinii* and *B. thetaiotaomicron* in YCFA medium supplemented with cecal small-molecule metabolites from LBC and AA. **D** The growth curve of *P. goldsteinii* in YCFA medium supplemented with cecal small-molecule metabolites from LBC orally administered with *B. thetaiotaomicron*. The data were analyzed using the Kruskal-Wallis test with Dunn’s multiple comparisons test for comparing groups. **A**–**C** (*n* = 5), (**D**) (*n* = 3).

Our data showed that the LBC has a high abundance of Bacteroidetes and strong polysaccharide-utilization capability. Specifically, MT treatment increased the relative abundance of *P. goldsteinii*. We investigated whether the microbial ecosystem characteristics of the LBC affected the engraftment of *P. goldsteinii* in the gut. *P. goldsteinii* was cultured separately in yeast extract-casein hydrolysate-fatty acid (YCFA) medium containing small metabolic molecules from the caecum of LBC and AA chickens. The small metabolites from both LBC and AA chickens supported the in vitro growth of *P. goldsteinii*, and LBC were more favorable for the growth of *P. goldsteinii* compared to AA (Fig. 7A).

We further investigated the potential mechanisms underlying the predominant colonization of *P. goldsteinii*. Previous studies confirmed that exclusive nutrient access is an important factor in controlling strain engraftment and abundance^23^. Therefore, we selected common polysaccharides from the diet and gut and analysed the carbohydrate-foraging capabilities of *P. goldsteinii* to determine whether the strong polysaccharide metabolic capacity of the LBC controls gut colonization. *P. goldsteinii* utilized glucose, galactose, and, partially mannose as energy substrates, but did not utilize other high-molecular-weight polysaccharides (Supplementary Fig S8).

We observed a positive correlation between *B. thetaiotaomicron* and *P. goldsteinii* abundance in the gut of the LBC (Supplementary Table S4). Therefore, we selected a typical polysaccharide degrader, *B. thetaiotaomicron*, which dedicates approximately 18% of its 6.26-Mb genome to the degradation of 88 PULs^24,25^. In BHI medium, we performed cocultures of *P. goldsteinii* and *B. thetaiotaomicron* or cultured one strain for 24 h before adding the other, but found no interaction between *P. goldsteinii* and *B. thetaiotaomicron* (Fig. 7B). When cultured in YCFA medium, caecal metabolites from both LBC and AA chickens supported the growth of *P. goldsteinii* and *B. thetaiotaomicron* (Fig. 7C). In coculture, the caecal metabolites from the LBC resulted in a higher OD_600_ during the growth plateau phase than those from AA chickens (Fig. 7C). We also observed significantly increased propionate contents (Supplementary Fig S9).

We orally administered *B. thetaiotaomicron* or saline to AA chicks daily from 1 to 21 days of age. *B. thetaiotaomicron* had no effect on the thickness of the mucus layer (Supplementary Fig S10A), the amount of ileal mucin (Supplementary Fig S10B), the levels of FITC-D in the plasma (Supplementary Fig S10C), or the levels of d-lactic acid, and DOA in the serum (Supplementary Fig S10D). 16S rRNA sequencing did not detect *P. goldsteini*i in the gut (Supplementary Fig S11), which may explain why *B. thetaiotaomicron* did not enhance intestinal mucosal barrier function. Notably, intestinal metabolites from chickens orally administered with promoted *B. thetaiotaomicron* the growth of *P. goldsteinii* in YCFA medium (Fig. 7D).

## Discussion

The intestinal mucosal barrier plays a crucial role in maintaining host homeostasis and its functioning is closely regulated by gut microbiota^1,26^. Numerous studies have shown that native poultry breeds possess unique physical characteristics that are related to their resilience^27^. Hence, it is important to screen for probiotics that can enhance intestinal function^28^. In this study, we identified *P. goldsteinii* as a key gut commensal that enhances intestinal mucosal barrier function through a novel mechanism: biotransformation of TCDCA into CDCA, which activates FXR signaling to promote mucin production. We further demonstrate that *Bacteroides* species create a metabolic niche facilitating *P. goldsteinii* colonization through cross-feeding. These findings have important implications for understanding microbiota-barrier interactions and developing targeted therapies for intestinal barrier dysfunction.

Probiotic manipulation is an important strategy for enhancing intestinal barrier function^29^. Some studies have reported that commensal bacteria play key roles in enhancing gut mucosal barrier function^30^. For example, *Akkermansia muciniphila* promotes mucin secretion by goblet cells to strengthen the mucosal barrier, and *B. thetaiotamicron* metabolically produces acetate to enhance goblet cell differentiation and promote mucin secretion^31^. However, developing probiotics from the same species to improve host health remains a challenging task in the utilization of microbial resources. A growing body of evidence suggests that native poultry breeds have intestinal microbial resources that confer stronger intestinal barrier function and stress tolerance^16,18^. In this study, 16S rRNA gene amplicon sequencing and metagenomics showed that the caecum of the LBC harboured a high abundance of *Bacteroides* and *P. goldsteinii*, accompanied by increased mucus layer thickness and mucin content. Indeed, local chicken varieties are known to possess a higher Bacteroides-to-Firmicutes ratio, possibly owing to their diverse diet that includes a large amount of fibre, unlike that of industrially raised broilers^18,32^. Moreover, AA broilers exhibit reduced intestinal mucin density after hatching^33^. These findings suggest that *Bacteroides* and *P. goldsteinii* may be associated with the intestinal mucosal barrier.

FMT is a promising approach for promoting beneficial microbial interactions and intestinal function^34^. A recent study revealed that AA chickens exhibit high growth efficiency traits and optimized mucosal architecture following transplantation of Tibetan chicken gut microbiota^35^. However, transplanting the gut microbiota of AA chickens to Tibetan chickens did not alter the growth efficiency, despite changes in microbial diversity^35^. This indicates that cross-FMT technology achieved inter-breed complementary advantages, but its effectiveness is influenced by the maturity of the microbiota. In the present study, MT from native LBC to AA demonstrated that the gut microbiota of LBC plays a crucial role in enhancing intestinal barrier function, including the secretion of mucin, thickness of the mucus layer, and the intestinal permeability. In addition, MT increased the abundance of *Parabacteroides* in the cecum of AA chickens, which aligns with the finding that a higher colonization of *P. goldsteinii* was observed in the cecum of LBC. Previous studies have confirmed that *P. goldsteinii* is a potential probiotic candidate for maintaining intestinal homeostasis^14^. In this study, administration of *P. goldsteinii* increased the thickness of the mucus layer and the secretion of mucin, and reduced the serum level of FITC-D, demonstrating its probiotic potential in enhancing intestinal mucosal barrier function.

Elucidating the mechanisms by which probiotics enhance intestinal barrier function still urgently needed, although previous studies have demonstrated the crucial function of gut microbiota in maintaining intestinal barrier homeostasis^36^. The probiotic effects of gut microbiota are primarily achieved through structural and metabolic factors^37,38^. Bile acids are important mediators between the gut microbiota and intestinal mucosal barrier^39,40^. Microbiota are key regulators of bile acid composition and the metabolism of primary bile acids into secondary bile acids by bile salt hydrolases^41,42^. However, few studies have focused on the effects of *Parabacteroides* spp. on host bile acid metabolism. Notably, our findings indicate that *P. goldsteinii* can bio-transform TCDCA into CDCA, which is similar to the previously reported function of *P. distasonis*^43^. Furthermore, CDCA activated intestinal epithelial FXR signaling to enhance intestinal mucosal barrier function. These results support the role of bile acids in FXR signaling and highlight the importance of CDCA in microbiota-mediated intestinal barrier function^7,38^. Thus, our findings reveal that the biotransformation of TCDCA into CDCA by *P. goldsteinii* plays a critical role in enhancing intestinal mucosal barrier function.

Microbial community structure and function are orchestrated by interactions among the microbiota^44,45^. Our results showed that the gut ecosystem of the LBC was more conducive to the colonization of *P. goldsteinii* than that of AA broilers. To understand the role of *P. goldsteinii*, it is important to unravel the underlying mechanisms. The LBC gut exhibited robust polysaccharide metabolic capabilities. This is consistent with the higher abundance of *Bacteroides* in the LBC gut, which are polysaccharide generalists. However, we found that *P. goldsteinii* could not directly metabolize polysaccharides. Cross-feeding—the utilization of metabolites from one bacterium by another under nutrient-limited conditions—is common and enhances the stability and diversity of microbial communities^45,46^. *Bacteroides* spp. are crucial nutrient providers by metabolizing polysaccharides into smaller molecules that support the colonization and growth of secondary consumers^20,47^. *B. vulgatus* degrades mucin to produce *N*-acetylglucosamine via *N*-acetylglucosaminidase. *N*-acetylglucosamine serves as a nutrient for *A. muciniphila*, and an increase in the abundance of *A. muciniphila* can ameliorate high-fat diet-induced metabolic syndrome and intestinal mucosal barrier injury^13^. *P. goldsteinii* and *B. thetaiotaomicron* exhibit a symbiotic relationship in the gut of LBC. *B. thetaiotaomicron* possesses a remarkable capacity for polysaccharide metabolism, with approximately 18% of its encoded genome dedicated to metabolizing polysaccharides^25^. Previous studies have identified *B. thetaiotaomicron* as a primary degrader of polysaccharides, engaging in intimate cross-feeding interactions with other commensal bacteriac^45^. In the co-culture of *P. goldsteinii* and *B. thetaiotaomicron*, small metabolites from the caecum of the LBC enhanced the interactions between *P. goldsteinii* and *B. thetaiotaomicron* compared to those from AA broilers. However, the administration of *B. thetaiotaomicron* to AA broilers did not enhance the gut colonization of *P. goldsteinii*. Considering the 16S rRNA results, the lower relative abundance of *P. goldsteinii* in the caeca of AA broilers may explain the poor mucosal barrier function. Moreover, we found that the caecal metabolites from *B. thetaiotaomicron*-administered AA broilers were more supportive of *P. goldsteinii* growth. Unfortunately, poultry diets are rich in a wide variety of polysaccharides, which are macromolecular substances that are difficult for us to detect; the specific small-molecule metabolites require further investigation. These findings suggest that the higher relative abundance of *Bacteroides* and robust polysaccharide metabolic capacity in the caeca of LBCs facilitate the colonization of *P. goldsteinii*, which in turn may improve the intestinal mucosal barrier.

In conclusion, this study identifies *P. goldsteinii* as a key commensal that enhances intestinal mucosal barrier function through TCDCA-to-CDCA biotransformation and subsequent FXR activation. Its colonization is facilitated by cross-feeding interactions with *Bacteroides*. Although we observed that small-molecule metabolites may be associated with polysaccharide metabolism of *Bacteroides*, further investigation is needed to elucidate the underlying cross-feeding mechanisms, which would provide a better ecological basis for the development of *P. goldsteinii* as a probiotic. These findings reveal a novel microbiota-bile acid-FXR axis regulating barrier integrity and suggest potential therapeutic strategies for barrier dysfunction-associated diseases.

## Methods

### Animals and experimental design

The live animal experiment and procedures in this study were approved by the Institutional Animal Care and Use Committee of Northwest A&F University (Permit No. NWAFAC 1008). All efforts were made to ensure the welfare of the animals and to provide a suitable environment. All birds were euthanized via CO₂ inhalation following an approved facility standard procedure. Chickens were placed at an appropriate density in a sealed chamber and exposed to regulated CO₂ for 30 s, with periodic re-flooding of the gas. After vocalization ceased, the chickens were left undisturbed for 2 minutes to confirm death. All birds had *ad libitum* access to water and feed throughout the experiments, and the diet formulation is shown in Supplementary Table S1.

Exp. 1 Comparison of gut microbiota landscape and intestinal mucosal barrier between LBC and AA broilers. To decipher the cecal microbiota landscape of LBC and AA chickens, a total of 122 LBC (the body weight reached 1.811 ± 0.232 kg, half male and half female) and 54 AA chickens (the body weight reached 3.058 ± 0.144 kg, half male and half female) were used in this study, aiming to depict the overall characteristics of the chicken cecal microbiota. 176 fresh cecal contents from 176 chickens were individually collected and immediately frozen in liquid nitrogen. Cecal microbial genomic DNA extraction was conducted as previously described^22^. In addition, plasma and intestinal tissue samples were collected to evaluate intestinal barrier function.

Exp. 2 Antibiotic administration. To investigate the correlation between microbiota and intestinal mucosal barrier function, antibiotic perturbation of the gut microbiota was performed in AA broilers. A total of 76 one-day-old male AA broilers were randomly divided into two groups. The control group (Con) contained 9 replicates with 4 broilers per replicate, and the antibiotic group (Anti) contained 10 replicates with 4 broilers per replicate. Broilers in Con were fed basal diet, and the Anti were fed basal diet added with 200 mg/kg vancomycin, ampicillin, metronidazole, and kanamycin for 21 days. One bird was selected from each replicate at 21 days of age. The serum, plasma, liver, ileal contents and cecal contents were collected. The cecal contents were harvested for subsequent DNA extraction.

Exp. 3 Microbiota transplantation experiments. To verify that the cecal microbiota of LBC can enhance the intestinal mucosal barrier function of AA broilers, we performed a cecal microbiota transplant from LBC to AA broilers. Cecal microbiota suspensions were obtained from healthy and non-antibiotic-fed male LBC (the body weight reached 2.5 kg). Microbiota suspensions were obtained and stored as reported previously^8^. In brief, chickens were sacrificed after which the cecal contents were collected and immediately transferred into an anaerobic operation apparatus. The cecal contents of LBC (1 g) were diluted in 10 mL of a sterile Ringer working buffer containing 20% glycerol (9 g/L sodium chloride, 0.4 g/L potassium chloride, 0.25 g/L of calcium chloride dehydrate and 0.05% (wt/vol) L-cysteine hydrochloride) in an anaerobic chamber (80%N_2_:10%CO_2_:10%H_2_). The cecal contents were suspended by thorough vortexing for 5 min and then centrifuged at 4 °C 2500 × *g* for 10 min. The suspension is stored at −80 °C degrees until later use. A total of 42 one-day-old male Arbor Acres broilers were randomly allocated into Control and microbiota suspension (MT) groups. Each group contained 7 replicates with 3 broilers per replicate. Chicks in two groups were orally inoculated with sterile PBS (Control) and cecal MT from LBC 0.5 mL/brid/day at 3 to 10 days of age and 1 mL/brid/day at 11 to 21 days of age, respectively. The serum, plasma, liver, ileal contents and cecal contents were collected. The cecal contents were harvested for subsequent DNA extraction.

Exp. 4 Oral gavage bacterial strains. *Parabacteroides goldsteinii* (ATCC BAA-1180) and *Bacteroides thetaiotaomicron* (ATCC 29148) were provided by the mingzhoubio in China. The *P. goldsteinii* and *B. thetaiotaomicron* were cultured in a brain–heart infusion (BHI) medium under anaerobic conditions at 37 °C.

To investigate whether *P. goldsteinii* enhances intestinal mucosal barrier function, we conducted an oral gavage experiment with *P. goldsteinii*. A total of 60 one-day-old male Arbor Acres broilers were randomly allocated into Control and *P. goldsteinii* groups. Each group contained 10 replicates with 3 broilers per replicate. Chicks in two groups were orally administrated with sterile BHI medium (1 mL) (Control) and *P. goldsteinii* suspension (1 mL, 10^8^ CFU/mL) (Pg) at 3 to 21 days of age, respectively. The serum, plasma, liver, ileal contents and cecal contents were collected. The cecal contents were harvested for subsequent DNA extraction. The ileal tissues were fixed to observe the tissue morphology.

To investigate whether *B. thetaiotaomicron* enriches *P. goldsteinii* through cross-feeding and thereby enhances intestinal mucosal barrier function, an oral gavage experiment with *B. thetaiotaomicron* was conducted. Oral gavage *B. thetaiotaomicron* experiment, a total of 56 one-day-old male Arbor Acres broilers were randomly allocated into Control and *B. thetaiotaomicron* groups. Each group contained 7 replicates with 4 broilers per replicate. Chicks in two groups were orally administrated with sterile BHI medium (1 mL) (Control) and *B. thetaiotaomicron* suspension (1 mL, 10^8^ CFU/mL) (Bt) at 3 to 21 days of age, respectively. The ileal tissues were fixed to observe the tissue morphology. The cecal contents were harvested for subsequent DNA extraction.

### Assessment of intestinal permeability

The concentration of serum DOA and d-lactic acid was determined by using the reagent kit (Nanjing Jiancheng Technology Co., Ltd., Nangjing, China). The concentration was measured according to the manufacturer’s instructions.

### Thickness measurements of the ileal mucus layer

The ileal tissues were freshly harvested from chickens and immediately preserved in Carnoy’s fixative with slight modification to a previous protocol^23^. The ileum was fixed in Carnoy’s solution for 4 h and then transferred to the fresh Carnoy’s solution for 4 h. The ileum was then washed twice in cold dry methanol for 2 h, placed in cassettes and stored in fresh dry methanol at 4 °C. The methanol-stored ileal tissues were embedded in paraffin and thin sections (~5 μm) were cut. Alcian blue staining was carried out according to the following protocol: 1) deparaffinization at 65 °C for more than 4 h, 2) dehydration with different alcohol concentration gradients for 2 min (100%, 95%, 90%, 80%, 70%), 3) Alcian blue solution for 25 min, 4) washing in running water for 1 min, 5) dehydration with 95% and absolute alcohol for 4 min, 6) cleanout in xylene and cover with coverslip. To measure the thickness of ileal mucus layer, the light microscope (Olympus Corporation, Tokyo, Japan) coupled with image processing software (Image J 1.53) were used to measure the thickness of mucus layer by selecting five complete mucus layer units.

### Histological analysis

For histological analysis of ileal tissue, it was measured as previously described^22^. Briefly, post Carnoy’s fixation, the ileal samples were routinely dehydrated, and then ileal tissues were embedded in paraffin and thin sections (~5 μm) were cut. Quantification of ileal mucin density was identified by Alcian blue-periodic acid Schiff (AB-PAS) following the manufacture’s instruction (Servicebio, Wuhan, China). The counts of mucin were measured in 5 randomly chosen intact villus per section with a light microscope (Olympus Corporation, Tokyo, Japan).

### 16S rRNA gene amplicon sequencing and data analysis

Cecal microbial genomic DNA extraction and 16S rRNA gene amplicon sequencing were set according to previously reported methods^22^. Briefly, total DNA was extracted from 174 cecal contents using a E.Z.N.A. soil DNA Kit (Omega Bio-tek, Norcross, GA, U.S.) according to manufacturer’s protocols. The quality of the extracted DNA was examined by electrophoresis in 1% (wt/vol) agarose gels. The bacterial V3-V4 region was amplified using the following primers: F, 5’-ACTCCTACGGGAGGCAGCAG-3’ and R, 5’-GGACTACHVGGGTWTCTAAT-3’. Highthroughput sequencing was conducted on the Illumina MiSeq platform (Illumina, San Diego, USA) according to the standard protocols. Raw Sequence data were analyzed with QIIME2 platform^48^. Quality control and denoising were performed using DADA2 with default parameters to generate Amplicon sequence variants (ASVs)^49^. The principal coordinate analysis (PCoA) was performed using weighted Bray-Curtis distance and Unifrac distance metrics. α-diversity was calculated on the free online platform of Microeco Tech Co., Ltd. (Shenzhen, China, https://bioincloud.tech/task-list). Differential taxa were identified by linear discriminant analysis effect size (LEfSe) analysis.

### Metagenomics sequencing and data analysis

Microbial genomic DNA was extracted from 34 cecal samples using cetyltrimethylammonium bromide method. The metagenome libraries were constructed through specific preparation kits (Illumina) and sequenced on Illumina platforms with PE150 strategy, according to effective library concentration and data amount required. The raw data of bacteria in cecal samples were obtained by metagenomic sequencing using Illumina Novaseq high-throughput sequencing platform. The raw data were filtered using Kneaddata software, and the host genomic reads were trimmed using Bowtie2 software (v2.2.5)^50^. FastQC was used to detect the rationality and effect of quality control. The species contained in the samples were identified using Kraken2 and a self-built microbial database, and then the actual relative abundance of species in the samples was predicted using Bracken^51,52^. Based on species abundance table and functional abundance table, abundance clustering analysis. Use LefSe biomarker analysis and Dunn test analysis to excavate differences in species composition and functional composition between samples.

### In vivo intestinal permeability assay using FITC-dextran analysis

To measure fluorescein isothiocyanate (FITC)-dextran (4 kDa; Sigma), chickens were fasted for 6 h and then were orally gavaged with FITC-dextran (7 mg/kg body weight) 3 h before blood collection. The concentration of FITC-dextran was monitored using a fluorescence spectrophotometer with an excitation wavelength of 493 nm and emission wavelength of 525 nm. Serum from chickens that had not received FITC-dextran was used as back-ground control^24^.

### Bile acids quantification in plasma, liver and ileum samples

The bile acids from plasma, liver, and ileal contents were analyzed using liquid chromatography-mass spectrometry (LC-MS), as previously described^22^. Briefly, samples consisting of 0.2 mL of plasma, approximately 60 mg of liver tissue, and ileal contents were subjected to extraction using 0.6 mL of a methanol:water (1:1). The mixture was vortexed for 15 min and subsequently centrifuged at 13,000 × *g* for 15 min at 4 °C, and subsequently the supernatant was mixed with methanol:acetonitrile (2:8). The homogenized solution was vortex for an additional 15 min aand centrifuged again under the same conditions. The supernatant was filtered using 0.22 μm filter. Finally, the LC-MS (Qtrap 5500, AB SCIEX) analysis was performed. Standard curves were prepared for the quantification of target substances in the samples.

### Measurement of serum cytokines

The enzyme-linked immunosorbent assay (ELISA, Quanzhou Ruixin Biological Technology Co., LTD.) was used to measure the level of SIgA and SIgM form serum according to the manufacture’s instructions.

### RNA isolation and quantitative real‑time PCR (qRT‑PCR)

The procedures for the PCR reaction and the associated calculations were carried out in accordance with the methods outlined in a previous study^25^. The ileal mucosa was thoroughly homogenized, and the total RNA was isolated following the guidelines provided with the TRIzol reagent (AG21102, AG, Changsha, China). The quantification and assessment of RNA concentration and purity were conducted utilizing the NanoDrop 2000 spectrophotometer. For cDNA synthesis, 500 ng of RNA was reverse transcribed using the Evo M-MLV Reverse Transcriptase Kit (AG11707, AG, Changsha, China). Te mRNA expression was performed using the SYBR Green Premix Pro Taq HS qPCR Kit (AG11701, AG, Changsha, China) on the iCycler IQ5 (Bio-Rad, Hercules, CA, USA). The primer sequences in the study are listed in Supplementary Table S2 as previously description^22^.

### Determination of the conversion of TCDCA to CDCA

To evaluate whether *P. goldsteinii* converts TCDCA into CDCA, *P. goldsteinii* was co-cultured with 25 µM or 500 µM TCDCA in brain–heart infusion liquid medium under anaerobic conditions at 37 °C for 24 h. The concentrations of CDCA and TCDCA in the medium were quantified using liquid chromatography-mass spectrometry (LC-MS). Each treatment was performed with five replicates.

### Cell culture and treatment

The human colon epithelial carcinoma cell line HT29 was obtained from the American Type Culture Collection (ATCC). Cells were maintained in Dulbecco’s modified Eagle’s medium (DMEM) containing 10% fetal bovine serum (FBS) and 1% penicillin/streptomycin in a humidified incubator (5% CO_2_, 95% air, 37 °C). Cell treatment methods were based on previous studies^15,53^. Briefly, Cells were grown until 70 to 80% confluence and treated with 50 mM CDCA (Sigma-Aldrich) and 50 mM GUDCA (FXR inhibitor, Sigma-Aldrich) for 24 h. Furthermore, HT-29 cells were treated with 0.5 µg/mL Tunicamycin (TM, protein glycosylation inhibitor, Sigma-Aldrich) for 6 h, followed by treatment with CDCA and GUDCA.

### Western blot analysis

Cells were lysed with RIPA extraction buffer (Solarbio, China) containing a 1% protease inhibitor cocktail to harvest total cellular protein. The concentration of total protein was quantified by bicinchoninc acid (BCA) protein assay kit (Solarbio, China). Proteins were separated by sodium dodecyl sulfate-polyacrylamide gel electrophoresis gel and then transferred to polyvinylidene difluoride (PVDF) membranes (Millipore, USA). The membranes were blocked with 5% skimmed milk for 1 h and incubated with primary antibodies against MUC2 (Abcam, USA) and FXR (Abcam, USA) overnight at 4 °C. The membranes were washed and incubated with HRP-labeled secondary antibodies at room temperature for 2 h. Protein signals were detected with ECL Kit.

### Determination of growth curve of *P. goldsteinii* and *B. thetaiotaomicron*

To investigate the impact of cecal small-molecule metabolites from LBC and AA broilers on the growth of *P. goldsteinii*, five healthy male AA broilers and LBC were selected respectively. The cecal contents were collected from each bird under sterile conditions. The cecal content was homogenized in sterile Ringer’s working solution (containing 0.4 g KCl, 9 g NaCl, 0.25 g CaCl₂·2H₂O, and 0.5 g L-cysteine hydrochloride per liter of distilled water) at a ratio of 1:10 (w/v). The mixture was vortexed thoroughly and centrifuged at 3500 rpm for 10 min at 4 °C. The supernatant was sterilized under high-pressure conditions and filtered through a 0.25 μm membrane filter. The filtered supernatant was then added to Yeast Casitone Fatty Acid (YCFA) medium at a ratio of 1:10. *P. goldsteinii* was inoculated into the prepared medium and cultured under anaerobic conditions at 37 °C for 48 h. The growth curve of *P. goldsteinii* was subsequently measured.

To investigate the metabolic capability of *P. goldsteinii* on various polysaccharides. *P. goldsteinii* was cultured in YCFA medium supplemented respectively with 1% (w/v) cellobiose, glucose, soluble starch, rhamnose, pectin, galactose, glycosaminoglycans, heparin sodium, chondroitin sulfate, hyaluronic acid, fucose, xylose, glucuronic acid, arabinose, or mannose. The mediums were cultured under anaerobic conditions at 37 °C for 48 h. Growth curves were then measured.

To investigate the interaction between *P. goldsteinii* and *B. thetaiotaomicron*, we conducted co-culture experiments under anaerobic conditions at 37 °C for 48 h. The experimental groups were designed as follows: 1) *P. goldsteinii* monoculture; 2) *B. thetaiotaomicron* monoculture; 3) simultaneous co-culture of *P. goldsteinii* and *B. thetaiotaomicron* (Bt.Pg); 4) sequential culture with *P. goldsteinii* inoculated 24 h prior to *B. thetaiotaomicron* (Pg-Bt); and 5) sequential culture with *B. thetaiotaomicron* inoculated 24 h prior to *P. goldsteinii* (Bt-Pg). Bacterial growth curves were monitored throughout the incubation period in BHI medium.

To investigate the interaction between *P. goldsteinii* and *B. thetaiotaomicron* under the influence of cecal small-molecule metabolite from LBC and AA broilers, we conducted co-culture experiments using YCFA medium under anaerobic conditions at 37 °C for 48 h. Experimental groups were designed as follows: 1) *B. thetaiotaomicron* monoculture in YCFA (Bt); 2) *P. goldsteinii* monoculture in YCFA (Pg); 3) co-culture of *B. thetaiotaomicron* and *P. goldsteinii* in YCFA (Bt-Pg); 4) *B. thetaiotaomicron* monoculture in YCFA containing AA cecal small-molecule metabolite (AA-Bt); 5) *P. goldsteinii* monoculture in YCFA containing AA cecal small-molecule metabolite (AA-Pg); 6) co-culture of *B. thetaiotaomicron* and *P. goldsteinii* in YCFA containing AA cecal small-molecule metabolite (AA-Bt-Pg); 7) *B. thetaiotaomicron* monoculture in YCFA containing LBC cecal small-molecule metabolite (LBC-Bt); 8) *P. goldsteinii* monoculture in YCFA containing LBC cecal small-molecule metabolite (LBC-Pg); 9) co-culture of *B. thetaiotaomicron* and *P. goldsteinii* in YCFA containing LBC cecal metabolite fractions (LBC-Bt-Pg). Growth curves were then measured.

To investigate the impact of *B. thetaiotaomicron*-induced metabolic niche on the growth of *P. goldsteinii*, we conducted in vitro culture experiments using cecal metabolites derived from AA broilers. Five healthy 21-day-old male AA broilers were randomly selected from both the control group and *B. thetaiotaomicron*-administrated group for cecal metabolite collection. *P. goldsteinii* was inoculated into YCFA medium containing these metabolites and cultured under anaerobic conditions at 37 °C for 48 h. Growth curves were then measured.

### Short-chain fatty acid profiling

Short-chain fatty acids (SCFAs) in the supernatant of the culture medium were quantified as reported previously [7]. Briefly, 1 mL of culture medium supernatant was mixed with 1 mL of cold saline homogenate and centrifuged at 13,000 × *g* for 10 min at 4 °C. The resulting supernatant was then deproteinized by adding 0.4 mL of metaphosphate. After incubation at 4 °C for 4 h, the mixture was centrifuged again under the same conditions (13,000 × *g*, 10 min, 4 °C). Subsequently, 0.2 mL of crotonic acid was added to the supernatant, and the mixture was filtered through a 0.45 μm membrane filter. The concentration of SCFAs in the processed samples was quantified using gas chromatography-mass spectrometry (GC-MS) on an Agilent 6890 system (Agilent Technologies, Santa Clara, CA, USA).

### Statistical analysis

Statistical analyses were performed using GraphPad Prism software. For statistical analysis of the two groups, intergroup differences were assessed using two-tailed unpaired Student’s *t* tests. For statistical analysis of more than two groups, the data were analyzed using the Kruskal‒Wallis test with Dunn’s multiple comparisons test for comparing groups. Detailed descriptions of the statistical methods are provided in the figure legends. Statistical significance was set at *P* < 0.05, which is indicated as follows: *P < 0.05, **P < 0.01.

## Supplementary information

Supplementary Information

Supplementary Table 4.

## Data Availability

The sequencing data were deposited in NCBI BioProject under accession numbers PRJNA1228982 (16S rRNA data for LBC and AA chickens), PRJNA1228986 (metagenomic data for LBC and AA chickens), PRJNA1228328 (antibiotic-treated chickens), PRJNA1228335 (microbiota transplantation experiments), and PRJNA1228307 (*B. thetaiotaomicron* gavage trials).
