# Supplementary material for: Gut commensal Parabacteroides goldsteinii-derived CDCA mediates FXR signalling to improve mucosal barrier function
Source: NPJ Biofilms Microbiomes. 2026 Jun 6;12:171. doi: 10.1038/s41522-026-01035-2 (PMC13562522; doi:10.1038/s41522-026-01035-2)
Supplement: Supplementary file 1 [file 41522_2026_1035_MOESM1_ESM.docx]

**Supplementary Information for**

**Gut commensal *Parabacteroides goldsteinii*-derived CDCA mediates FXR signalling to improve mucosal barrier function**

Jiantao Yang^1#^, Kailong Qin^1#^, Qianggang Wang^1^, Wei Guo^2^, Xiaojun Yang^1*^

Supplementary Figure 1	2

Supplementary Figure 2	3

Supplementary Figure 3	4

Supplementary Figure 4	5

Supplementary Figure 5	6

Supplementary Figure 6	7

Supplementary Figure 7	8

Supplementary Figure 8	9

Supplementary Figure 9	10

Supplementary Figure 10	11

Supplementary Figure 11	12

Supplementary Table 1	13

Supplementary Table 2	15

Supplementary Table 3	16

Supplementary Figure 1. Functional prediction of cecal microbiota in LBC and AA based on metagenomics. (A) KEGG signaling pathways related to carbohydrate metabolism. (B) KEGG signaling pathways related to glycan metabolism. (C) KEGG signaling pathways related to animal-derived glycan metabolism. (D) KEGG signaling pathways related to polysaccharide metabolism at the KO level.

Supplementary Figure 2. Antibiotic-induced intestinal microbiota dysbiosis. (A) PCoA of the gut microbiome composition on the OTU level in chickens. (B) Analysis of α-diversity index of richness (Chao) and community diversity (Shannon). (C) Relative abundance of Bacteroidetes and Proteobacteria. (D) Relative abundance of top 19 genera.

Supplementary Figure 3. Intestinal mucosal barrier injury caused by microbial dysbiosis. (A, B) Representative images of Alcian blue-stained ileal sections, and the thickness of mucus layer. (C, D) Representative images of AB-PAS-stained ileal sections, and the number of mucin. (E-F) Intestinal permeability serum FITC-dextran levels, and plasma diamine oxidase activities.

Supplementary Figure 4. Effects of microbiota transplantation from LBC to AA broilers on gut microbiota. (A) Analysis of α-diversity index. (B) PCoA of the gut microbiome composition on the OTU level. (C) Relative abundance of top 19 genera.

Supplementary Figure 5. The impact of oral administration of *P. goldsteinii* on ileal bile acids. CDCA, Chenodeoxycholic acid; GCDCA, Glycochenodeoxycholic acid; CA, Cholic acid; TCA, Taurocholic Acid; GCA, Glycocholic acid; DCA, Deoxycholate acid; TDCA, Taurodeoxycholic acid; UDCA, Ursodeoxycholic acid; LCA, Lithocholate; TLCA, Taurolithocholic acid; TCDCA, Taurochenodeoxycholic acid.

Supplementary Figure 6. Effects of microbiota transplantation from LBC to AA broilers on ileal bile acids. CDCA, Chenodeoxycholic acid; GCDCA, Glycochenodeoxycholic acid; CA, Cholic acid; TCA, Taurocholic Acid; GCA, Glycocholic acid; DCA, Deoxycholate acid; TDCA, Taurodeoxycholic acid; UDCA, Ursodeoxycholic acid; LCA, Lithocholate; TLCA, Taurolithocholic acid; TCDCA, Taurochenodeoxycholic acid.

Supplementary Figure 7. The impact of antibiotic-induced gut microbiota disruption on bile acids. CDCA, Chenodeoxycholic acid; GCDCA, Glycochenodeoxycholic acid; CA, Cholic acid; TCA, Taurocholic Acid; GCA, Glycocholic acid; DCA, Deoxycholate acid; TDCA, Taurodeoxycholic acid; UDCA, Ursodeoxycholic acid; LCA, Lithocholate; TLCA, Taurolithocholic acid; TCDCA, Taurochenodeoxycholic acid.

Supplementary Figure 8. The growth curve of *P. goldsteinii* in YCFA medium supplemented with various polysaccharides.

Supplementary Figure 9. The content of SCFAs produced by *P. goldsteinii* and *B. thetaiotaomicron* in YCFA medium supplemented with cecal small molecule metabolites from LBC and AA.

Supplementary Figure 10. The impact of oral administration of *B. thetaiotaomicron* on intestinal mucosal barrier function. (A) Representative images of Alcian blue-stained ileal sections, and the thickness of mucus layer. (B) Representative images of AB-PAS-stained ileal sections, and the number of mucin. (C) Serum FITC-dextran levels. (D) Plasma diamine oxidase activities. (D) Plasma lactic acid levels.

Supplementary Figure 11. The impact of oral administration of *B. thetaiotaomicron* on the gut microbiota (relative abundance of top 19 genera).

Supplementary Table 1. Ingredients and nutrient composition of experiment diets (as-fed basis)

| Ingredients, % |  |
| --- | --- |
| Corn | 57.27 |
| Soybean meal | 35.2 |
| Cottonseed meal | 2.00 |
| Soybean oil | 2.00 |
| NaCl | 0.36 |
| Limestone | 2.00 |
| Calcium hydrogen phosphate | 0.30 |
| Choline chloride | 0.05 |
| L-lysine hydrochloride | 0.14 |
| Mineral premix^a^ | 0.30 |
| Phytase | 0.10 |
| Vitamin premix^b^ | 0.03 |
| DL-methionine | 0.25 |
| Total | 100 |
| Analyzed nutrient level |  |
| Metabolic energy, kcal/kg | 2950 |
| Dry matter | 86.41 |
| Crude fiber | 3.02 |
| Crude protein | 21.91 |
| Ash | 6.26 |
| Total phosphorus | 0.61 |
| Calcium | 0.96 |
| Methionine | 0.57 |
| Lysine | 1.21 |

^a^Mineral premix provided the following per kg of the diet: Mn, 80 mg; I, 0.40 mg; Fe, 80 mg; Cu, 10 mg; Zn, 70 mg; Se, 0.30 mg

^b^Vitamin premix provided the following per kg of the diet: vitamin A, 250,000 IU; vitamin D, 50,000 IU; vitamin K3, 53 mg; vitamin B1, 40 mg; vitamin B2, 120 mg; vitamin B12, 0.50 mg; vitamin E, 600 IU; biotin, 0.65 mg; folic acid, 25 mg; pantothenic acid, 240 mg; niacin, 1,000 mg

Supplementary Table 2. Forward and Reverse primer sequences for PCR analysis

| Genes | Forward Primer | Reverse Primer |
| --- | --- | --- |
| *β-actin* | ATTGTCCACGCAAATGCTTC | AAATAAAGCCATGCCAACTCGTC |
| *MUC2* | TTCATGATGCCTGCTCTTGTG | CCTGAGCCTTGGTACATTCTTG |

Abbreviations: *β-actin*, the internal reference gene beta actin, *MUC2,* mucin 2.

Supplementary Table 3. KEGG signaling pathways related to polysaccharide metabolism at the KO level.

| KO ID | Definition | Gene name | EC No. |
| --- | --- | --- | --- |
| K02744 | PTS system, N-acetylgalactosamine-specific IIA component | agaF | EC:2.7.1.- |
| K02745 | PTS system, N-acetylgalactosamine-specific IIB component | agaV | EC:2.7.1.- |
| K02746 | PTS system, N-acetylgalactosamine-specific IIC component | agaW | － |
| K02747 | PTS system, N-acetylgalactosamine-specific IID component | agaE | － |
| K02759 | PTS system, cellobiose-specific IIA component | celC | EC:2.7.1.196 |
| K02760 | PTS system, cellobiose-specific IIB component | celA | EC:2.7.1.196 |
| K02761 | PTS system, cellobiose-specific IIC component | celB | － |
| K02770 | PTS system, fructose-specific IIC component | fruA | － |
| K02793 | PTS system, mannose-specific IIA component | manX | EC:2.7.1.191 |
| K02794 | PTS system, mannose-specific IIB component | manX | EC:2.7.1.191 |
| K02795 | PTS system, mannose-specific IIC component | manY | － |
| K02796 | PTS system, mannose-specific IID component | manZ | － |
| K02804 | PTS system, N-acetylglucosamine-specific IIC component | nagE | － |
| K02810 | PTS system, sucrose-specific IIC component | scrA | － |

Fig 6
